# Update: Influenza Activity in the United States During the 2017–18 Season and Composition of the 2018–19 Influenza Vaccine

**DOI:** 10.15585/mmwr.mm6722a4

**Published:** 2018-06-08

**Authors:** Rebecca Garten, Lenee Blanton, Anwar Isa Abd Elal, Noreen Alabi, John Barnes, Matthew Biggerstaff, Lynnette Brammer, Alicia P. Budd, Erin Burns, Charisse N. Cummings, Todd Davis, Shikha Garg, Larisa Gubareva, Yunho Jang, Krista Kniss, Natalie Kramer, Stephen Lindstrom, Desiree Mustaquim, Alissa O’Halloran, Wendy Sessions, Calli Taylor, Xiyan Xu, Vivien G. Dugan, Alicia M. Fry, David E. Wentworth, Jacqueline Katz, Daniel Jernigan

**Affiliations:** 1 Influenza Division, National Center for Immunization and Respiratory Diseases, CDC.

The United States 2017–18 influenza season (October 1, 2017–May 19, 2018) was a high severity season with high levels of outpatient clinic and emergency department visits for influenza-like illness (ILI), high influenza-related hospitalization rates, and elevated and geographically widespread influenza activity across the country for an extended period. Nationally, ILI activity began increasing in November, reaching an extended period of high activity during January–February, and remaining elevated through March. Influenza A(H3N2) viruses predominated through February and were predominant overall for the season; influenza B viruses predominated from March onward. This report summarizes U.S. influenza activity* during October 1, 2017–May 19, 2018.^†^

## Virus Surveillance

CDC receives influenza test results from public health and clinical laboratories located in all 50 states, Puerto Rico, and the District of Columbia through U.S. World Health Organization (WHO) Collaborating Laboratories and the National Respiratory and Enteric Virus Surveillance System (NREVSS). During October 1, 2017–May 19, 2018, clinical laboratories tested 1,210,053 specimens for influenza virus; 224,113 (18.5%) tested positive (Supplementary Figure 1, https://stacks.cdc.gov/view/cdc/54973), including 151,413 (67.6%) for influenza A and 72,700 (32.4%) for influenza B. Nationally, the percentage of clinical laboratory–tested specimens positive for influenza virus peaked for 5 consecutive weeks during January 13–February 10 (surveillance weeks 2–6) (range = 26.1%–26.9%). Regionally,^§^ the week of peak clinical laboratory influenza positivity varied, ranging from the week ending December 30 (week 52) to the week ending February 17 (week 7).

Public health laboratories tested 98,446 specimens during October 1, 2017–May 19, 2018; 53,790 (54.6%) were positive for influenza viruses, including 38,303 (71.2%) positive for influenza A and 15,487 (28.8%) for influenza B (Supplementary Figure 2, https://stacks.cdc.gov/view/cdc/54974). Among the 37,681 seasonal influenza A viruses subtyped, 31,977 (84.9%) were influenza A(H3N2), and 5,704 (15.1%) were influenza A(H1N1)pdm09. Influenza B lineage information was available for 11,950 (77.2%) influenza B viruses; 10,612 (88.8%) were B/Yamagata lineage, and 1,338 (11.2%) were B/Victoria lineage. Whereas influenza A(H3N2) viruses accounted for the majority of circulating viruses, the proportion of influenza A viruses subtyped as A(H1N1)pdm09 ranged regionally from 9.0% in the central United States to approximately 24% in the northwestern and southeastern United States. Influenza B viruses were more commonly reported than were influenza A viruses from early March to late May (weeks 9–20). The proportion of influenza B viruses reported regionally ranged from 23.0% in the Midwest to 40.6% in the northwestern United States.

Among 47,121 (87.6%) patients who tested positive for seasonal influenza virus by public health laboratories and for whom age data were available, 3,802 (8.1%) were aged 0–4 years; 11,550 (24.5%), 5–24 years; 15,597 (33.1%), 25–64 years; and 16,172 (34.3%), ≥65 years. Influenza A(H3N2) viruses predominated among all age groups, ranging from 51.2% of viruses among persons aged 5–24 years to 70.0% among persons aged ≥65 years. The largest proportion of reported influenza B viruses (36.5%) occurred in persons aged 5–24 years.

## Antigenic and Genetic Characterization of Influenza Viruses

Public health laboratories participating as U.S. WHO collaborating laboratories submit a subset of influenza-positive respiratory specimens to CDC for virus characterization through three National Influenza Reference Centers in the California, New York, and Wisconsin state public health laboratories. CDC characterizes influenza viruses through genomic sequencing and antigenic characterization (using hemagglutination inhibition [HI] or neutralization assays). This process evaluates whether genetic changes in circulating viruses have led to antigenic drift away from the vaccine reference virus.

Influenza-positive specimens are sequenced using next-generation sequencing (NGS)^¶^ on the MiSeq System platform (Illumina), using genomic enrichment practices (*1*,*2*) adapted by CDC. Genomic data are analyzed to determine the genetic identity of circulating viruses and submitted to public databases (GenBank or GISAID EpiFlu).

CDC evaluates the antigenic similarity** between ferret antisera raised against reference viruses representing the recommended vaccine components of the Northern Hemisphere 2017–18 vaccine and circulating viruses isolated and propagated in mammalian cell culture. Since the 2014–15 season, many influenza A(H3N2) viruses propagated in mammalian cell culture have lacked sufficient hemagglutination titers for antigenic characterization using HI assays. Therefore, in addition to the use of the HI assay, a subset of influenza A(H3N2) viruses are antigenically characterized using a focus reduction assay (FRA) to assess the ability of various antisera to neutralize infectivity of the test viruses.

CDC has genetically characterized 3,329 influenza viruses collected since October 1, 2017, including 832 influenza A(H1N1)pdm09 viruses, 1,313 influenza A(H3N2) viruses, and 1,184 influenza B viruses. A subset of these viruses was also antigenically characterized.

Phylogenetic analysis of the hemagglutinin (HA) gene segments from 832 A(H1N1)pdm09 viruses collected since October 1, 2017, showed that all viruses belonged to subclade 6B.1 (Supplementary Figure 3, https://stacks.cdc.gov/view/cdc/54975). This has been the predominant HA clade in the United States since the 2015–16 season (*3*). Of the 736 A(H1N1)pdm09 viruses analyzed using HI assays, 735 (99.9%) were well inhibited (i.e., reacted at titers that were within fourfold of the homologous virus titer) by ferret antisera raised against cell culture–propagated 6B.1 virus A/Michigan/45/2015, the reference virus representing the A(H1N1)pdm09 vaccine virus for the 2017–18 Northern Hemisphere influenza season.

A total of 1,313 influenza A(H3N2) viruses were sequenced, and phylogenetic analysis of the HA gene segments indicated that multiple clades/subclades were cocirculating (Supplementary Figure 3, https://stacks.cdc.gov/view/cdc/54975), with 3C.2a predominating. Viruses with the 3C.2a HA emerged at the end of the 2013–14 season and have remained the predominant clade since the 2014–15 season (*4*), undergoing continued genetic diversification each season. Among 655 representative A(H3N2) viruses antigenically characterized by HI or FRA, 612 (93.4%) were well inhibited by ferret antisera raised against A/Michigan/15/2014 (3C.2a), a cell-propagated reference virus representing A/Hong Kong/4801/2014 (the A(H3N2) component of the 2017–18 Northern Hemisphere influenza vaccines). Only 6.6% of A(H3N2) viruses, the majority of which belonged to genetic clade 3C.3a, showed evidence of antigenic drift (i.e., had eightfold or greater reductions in HI or FRA titers compared with reference virus titers). In contrast to the 93.4% of A(H3N2) viruses that were well inhibited by ferret antisera raised against cell-propagated A/Michigan/15/2014, only 48.2% of viruses tested were well inhibited by ferret antiserum raised against the egg-propagated A/Hong Kong/4801/2014 reference virus representing the A(H3N2) vaccine component. A higher proportion (77.3%) of viruses tested were well inhibited by ferret antisera raised against egg-propagated A/Singapore/INFIMH-16–0019/2016 reference virus, representing the A(H3N2) component recommended for the 2018 Southern Hemisphere and the 2018–19 Northern Hemisphere influenza vaccines.

Phylogenetic analysis of 896 influenza B/Yamagata-lineage viruses showed that all HA gene segments belonged to clade Y3 (Supplementary Figure 3, https://stacks.cdc.gov/view/cdc/54975), which also predominated in the 2016–17 season (*5*). All 824 B/Yamagata lineage viruses that were antigenically characterized were antigenically similar to cell culture–propagated B/Phuket/3073/2013, the reference virus representing the B/Yamagata-lineage component of quadrivalent vaccines for the 2017–18 Northern Hemisphere influenza season.

The HA gene segment of 288 influenza B/Victoria-lineage viruses sequenced and phylogenetically analyzed belonged to genetic clade V1A, the same genetic clade as the vaccine reference virus, B/Brisbane/60/2008. However, 234 (81.3%) viruses had a six-nucleotide deletion in the HA gene segment (encoding amino acids 162 and 163). Viruses like these, previously abbreviated as V1A-2Del and now designated as V1A.1, were first reported during the 2016–17 season (*5*). Among 270 antigenically characterized influenza B/Victoria viruses, only 53 (19.6%) were antigenically similar to cell culture–propagated B/Brisbane/60/2008, the reference virus representing the B/Victoria lineage component of 2017–18 Northern Hemisphere vaccines. All 217 B/Victoria viruses that were poorly inhibited by antisera raised to B/Brisbane/60/2008 (i.e., had eightfold or greater reductions in HI titers compared with reference virus titers) had the V1A.1 HA segment. Circulating B/Victoria lineage V1A.1 viruses were well inhibited by ferret antisera raised against B/Colorado/06/2017, a V1A.1 reference virus representing the influenza B component recommended for the 2018–19 Northern Hemisphere influenza vaccine.

## Antiviral Susceptibility of Influenza Viruses

CDC tested 4,619 influenza viruses from the United States collected since October 1, 2017, for resistance to the influenza neuraminidase inhibitor antiviral medications recommended for use against seasonal influenza (oseltamivir, peramivir, and zanamivir). Among 1,147 influenza A(H1N1)pdm09 viruses tested for oseltamivir and peramivir susceptibility, 11 (1.0%) were resistant to both drugs and contain a known marker of resistance in the neuraminidase gene segment (H275Y). Among 786 influenza A(H1N1)pdm09 viruses also tested for zanamivir susceptibility, no resistant viruses were detected. All 2,354 influenza A(H3N2) viruses tested for oseltamivir and zanamivir susceptibility were susceptible to both medications. No peramivir-resistant viruses were detected among 1,248 A(H3N2) viruses tested. All 1,118 influenza B viruses tested were susceptible to all three medications. High levels of resistance to the adamantanes (amantadine and rimantadine) persist among influenza A viruses. Adamantane drugs are not recommended for use against influenza at this time.

## Composition of the 2018–19 Influenza Vaccine

The Food and Drug Administration’s Vaccines and Related Biologic Products Advisory Committee recommended that the 2018–19 trivalent vaccine to be used in the United States contain an A/Michigan/45/2015 A(H1N1)pdm09-like virus, an A/Singapore/INFIMH-16–0019/2016 A(H3N2)-like virus, and a B/Colorado/06/2017-like (B/Victoria lineage) virus (*6*). The quadrivalent vaccine recommendation included the trivalent vaccine viruses as well as a B/Phuket/3073/2013-like (B/Yamagata lineage) virus. The B component recommendation represents a change in the influenza B/Victoria lineage component recommended for the 2017–2018 Northern Hemisphere and 2018 Southern Hemisphere influenza vaccines. The B component change was made because of the increasing global circulation of an antigenically drifted B/Victoria lineage virus (V1A.1) (*7*). The A(H3N2) recommendation represents an update to the 2017–2018 Northern Hemisphere vaccines but is the same A(H3N2) virus recommended for the 2018 Southern Hemisphere vaccine. The decision to update the A(H3N2) component was not made to address antigenic drift, but rather because the egg-propagated A/Singapore vaccine virus is antigenically more similar to circulating viruses than the egg-propagated A/Hong Kong vaccine virus recommended for the Northern Hemisphere 2017–2018 vaccine. Vaccine recommendations were based on factors including global influenza virologic and epidemiologic surveillance, genetic characterization, antigenic characterization, and the candidate vaccine viruses that are available for production.

## Outpatient Illness Surveillance

**FIGURE 1:**
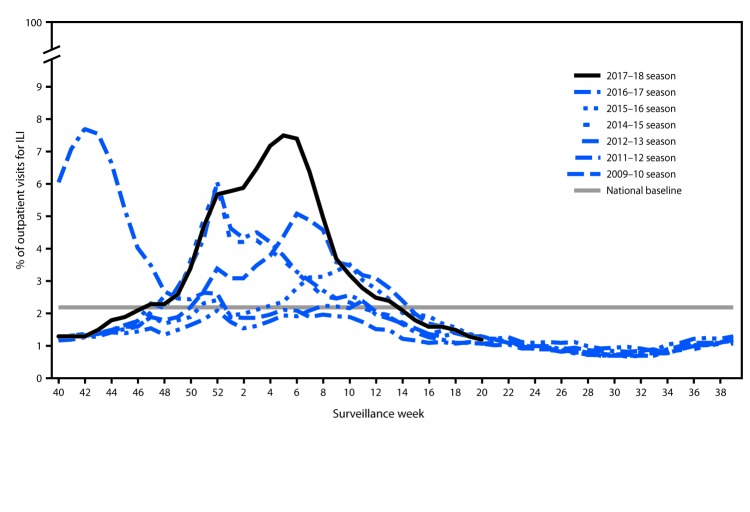
Percentage of outpatient visits for influenza-like illness (ILI)* reported to CDC, by surveillance week — U.S. Outpatient Influenza-Like Illness Surveillance Network (ILINET), national summary, United States, 2017–18^†^ influenza season and selected previous influenza seasons * Defined as fever (temperature of ≥100°F [≥37.8°C], oral or equivalent) and cough or sore throat, without a known cause other than influenza. ^†^ As of June 1, 2018.

Nationally, the weekly percentage of outpatient visits for ILI^††^ to health care providers participating in the United States Outpatient Influenza-like Illness Surveillance Network (ILINet) was at or above the national baseline^§§^ level of 2.2% for 19 consecutive weeks (weeks 47–13) during the 2017–18 season (Figure 1). The percentage of outpatient ILI visits exceeded 7.0% for three consecutive weeks, peaking at 7.5% during the week ending February 3, 2018 (week 5). During the 2012–13 through 2016–17 seasons, peak weekly percentages of outpatient ILI visits ranged from 3.6%–6.1% and remained at or above baseline levels for an average of 16 weeks (range = 11–20 weeks).

ILINet data are used to produce a weekly jurisdiction-level measure of ILI activity,^¶¶^ ranging from minimal to high. For the weeks ending December 30, 2017–February 24, 2018, approximately half of the 53 jurisdictions experienced high ILI activity each week, with the highest number (46; 87%) during the weeks ending January 27–February 10, 2018 (weeks 4–6). During the past 5 seasons, the highest number of jurisdictions experiencing high ILI activity in a single week ranged from 16 (30%) during the 2015–16 season to 31 (58%) during the 2012–13 season.

## Geographic Spread of Influenza Activity

State and territorial epidemiologists report the geographic distribution of influenza in their jurisdictions*** through a weekly influenza activity code.^†††^ During the 2017–18 season, the peak number of jurisdictions reporting widespread activity in a single week was 50 (93%); this occurred for 3 consecutive weeks (weeks ending January 6, 13, and 20, 2018). During the previous 5 influenza seasons, the peak number of jurisdictions reporting widespread activity in a single week during each season has ranged from 41 (76%) (2015–16 season) to 48 (89%) (2012–13 season).

## Influenza-Associated Hospitalizations

**FIGURE 2:**
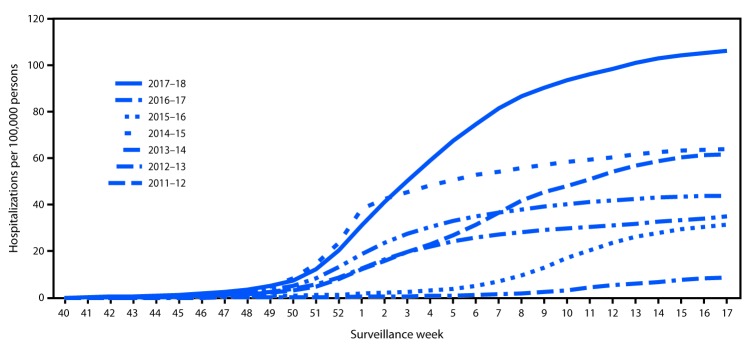
Cumulative rates of hospitalizations for laboratory-confirmed influenza by season and surveillance week — FluSurv-NET,* United States, 2011–12 through 2017–18 influenza seasons^†^ * FluSurv-NET conducts population-based surveillance for laboratory-confirmed influenza-associated hospitalizations in children aged <18 years (since the 2003–04 influenza season) and adults aged ≥18 years (since the 2005–06 influenza season). FluSurv-NET covers over 70 counties in the 10 Emerging Infections Program states (California, Colorado, Connecticut, Georgia, Maryland, Minnesota, New Mexico, New York, Oregon, and Tennessee) and three additional Influenza Hospitalization Surveillance Project states (Michigan, Ohio, and Utah). ^†^ As of June 1, 2018.

CDC monitors hospitalizations associated with laboratory-confirmed influenza infections through the Influenza Hospitalization Surveillance Network (FluSurv-NET),^§§§^ which covers approximately 27 million persons (9% of the U.S. population). During October 1, 2017–April 30, 2018, a total of 30,453 laboratory-confirmed influenza-related hospitalizations were reported (cumulative incidence for all age groups = 106.6 per 100,000 population) (Figure 2). The overall peak occurred during the week ending January 6, 2018 (week 1). The hospitalization rate was highest among persons aged ≥65 years, who accounted for approximately 58% of reported influenza-associated hospitalizations. By age group, the cumulative hospitalization rate was 74.3 per 100,000 population among children aged 0–4 years, 20.2 among children and adolescents aged 5–17 years, 32.6 among adults aged 18–49 years, 115.7 among adults aged 50–64 years, and 460.9 among adults aged ≥65 years. Among all influenza-associated hospitalizations, 22,023 (72.3%) were for influenza A virus infections, 8,226 (27.0%) for influenza B virus infections, 116 (0.4%) for influenza A virus and influenza B virus coinfections, and 88 (0.3%) for an influenza virus for which no type testing was done. Among 7,352 patients for whom influenza A subtype information was available, 6,163 (83.8%) were infected with influenza A(H3N2) viruses, and 1,189 (16.2%) were infected with influenza A(H1N1)pdm09 viruses.

Complete medical chart abstraction data in FluSurv-NET will not be finalized until later in 2018; however, as of June 1, 2018, data were available for 7,584 (24.9%) hospitalized adults and children with laboratory-confirmed influenza. Among 6,910 hospitalized adults with information on underlying medical conditions, 6,385 (92.4%) had at least one reported underlying medical condition that placed them at high risk^¶¶¶^ for influenza-associated complications. The most commonly reported underlying medical conditions among adults were cardiovascular disease (46.3%), metabolic disorders (43.3%), obesity (36.5%), and chronic lung disease (29.6%). Among 674 hospitalized children with such information, 382 (56.7%) had at least one underlying medical condition; the most commonly reported were asthma (23.4%), neurologic disorder (15.4%), and obesity (10.7%). Among 609 hospitalized women aged 15–44 years with information on pregnancy status, 187 (30.7%) were pregnant.

## Pneumonia and Influenza-Associated Mortality

CDC tracks pneumonia and influenza (P&I)–attributed deaths through CDC’s National Center for Health Statistics (NCHS) Mortality Reporting System. The percentages of deaths attributed to P&I are released 2 weeks after the week of death to allow for collection of sufficient data to produce a stable P&I mortality percentage. During the 2017–18 season, based on data from NCHS, the proportion of deaths attributed to P&I was at or above the epidemic threshold**** for 16 consecutive weeks during the weeks ending December 23, 2017–April 7, 2018 (weeks 51–14). Nationally, mortality attributed to P&I exceeded 10.0% for 4 consecutive weeks, peaking at 10.8% during the week ending January 20, 2018 (week 3).

## Severity Assessment

**FIGURE 3:**
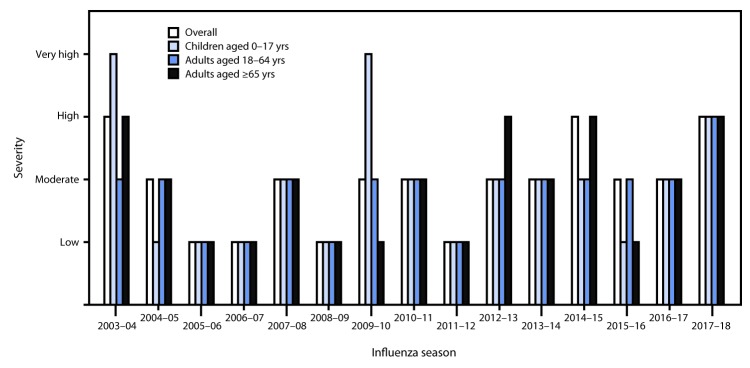
Influenza season severity classification,* by age group and season — United States, 2003–04 through 2017–18 seasons^†^ * CDC began using a new method in 2017 to classify influenza season severity using three indicators: the percentage of visits to outpatient clinics for influenza-like illness (ILI) from ILINet, the rates of influenza-associated hospitalizations from FluSurv-Net, and the percentage of deaths resulting from pneumonia or influenza from the National Center for Health Statistics. This method was applied retrospectively, going back to the 2003–04 influenza season. https://www.cdc.gov/flu/professionals/classifies-flu-severity.htm. ^†^ As of June 1, 2018.

In 2017, CDC began using a new methodology to classify influenza season severity using three indicators: 1) the percentage of visits to outpatient clinics for ILI from ILINet, 2) the rates of influenza-associated hospitalizations from FluSurv-Net, and 3) the percentage of deaths resulting from pneumonia or influenza from NCHS (*8*). This approach uses data from past influenza season indicators to calculate three intensity thresholds (ITs) (additional information is available at https://www.cdc.gov/flu/professionals/classifies-flu-severity.htm). These ITs help assess the historic chance that surveillance system data will exceed a certain threshold. CDC then classifies the severity of the current influenza season by determining which IT was crossed by at least two of the peak values from the above indicators. Based on this method, the severity of the 2017–18 season was classified as high severity overall and high severity for each age group (children and adolescents, adults, and older adults). This is the first time that each age group was classified as high in the same season, in a retrospective analysis going back to the 2003–04 season (Figure 3).

## Influenza-Associated Pediatric Mortality

CDC monitors pediatric influenza-associated deaths through the Influenza-Associated Pediatric Mortality Surveillance System. As of June 1, 2018, a total of 171 laboratory-confirmed influenza-associated pediatric deaths during the 2017–18 season had been reported to CDC from Chicago, New York City, and 41 states. Of these deaths, 36 (21%) were associated with infection with an influenza A(H3N2) virus, 31 (18%) with an influenza A(H1N1)pdm09 virus, 36 (21%) with an influenza A virus for which no subtyping was performed, 64 (37%) with an influenza B virus, two (1%) with an influenza A and B coinfection, and two (1%) with an influenza virus for which the type was not determined. The mean age of the pediatric deaths reported this season was 7.1 years (range = 8 weeks–17 years); 97 (57%) children died after admission to the hospital. Among the 154 children with a known medical history, 79 (51%) had at least one underlying medical condition recognized by the Advisory Committee on Immunization Practices (ACIP) as placing them at high risk for influenza-related complications. Among the 138 children who were eligible for influenza vaccination (age ≥6 months at date of onset) and for whom vaccination status was known, 30 (22%) had received at least 1 dose of influenza vaccine before illness onset (28 were fully vaccinated according to 2017 ACIP recommendations, and two had received 1 of 2 recommended doses). Since influenza-associated pediatric mortality became a nationally notifiable condition in 2004, the total number of influenza-associated pediatric deaths per season has ranged from 37 during the 2011–12 season to 171 during the 2012–13 season, excluding the 2009 pandemic, during which 358 pediatric deaths were reported to CDC during April 15, 2009–October 2, 2010.

## Discussion

The 2017–18 influenza season was a high severity, A(H3N2)-predominant season. In 2017, CDC began using a new methodology to classify seasonal severity and applied the methodology to the 2003–04 through 2016–17 seasons. The 2017–18 season is the third overall high severity season since 2003–04 and the first classified as high severity for all age groups (*8*). The peak percentage of outpatient visits for ILI was the third highest recorded since 1997–98, when ILINet was implemented. Mortality attributed to P&I remained above epidemic threshold for 16 consecutive weeks, peaking at 10.8%, the highest percentage reported since the 2014–15 season, when NCHS mortality data were first presented for routine influenza surveillance purposes. The cumulative hospitalization rate for laboratory-confirmed influenza for all ages combined and for the three adult age groups was the highest documented since the system expanded to include adults during the 2005–06 season. Although the hospitalization rates for children this season did not exceed the rates reported during the 2009 pandemic, they surpassed rates reported in previous high severity A(H3N2)-predominant seasons. These hospitalization rates are not adjusted for testing practices, which can vary from season to season; therefore, caution should be used when comparing hospitalization rates across seasons.

Influenza-associated pediatric mortality became a nationally notifiable condition in 2004. Excluding the 2009 pandemic, the previous highest number of pediatric deaths was reported during the 2012–13 season. The 171 pediatric deaths reported so far this season, approximately half in otherwise healthy children, equal the numbers reported during 2012–13 season. Although A(H3N2) was the predominant subtype circulating, there was substantial diversity in type and subtype of influenza infections leading to death in children. Less than one fourth (22%) of vaccine-eligible children who died from influenza this season had received influenza vaccine before illness onset.

Analysis of the influenza A(H3N2), A(H1N1)pdm09, and B/Yamagata lineage viruses showed that circulating viruses were antigenically similar to the cell-grown reference viruses representing the 2017–18 Northern Hemisphere influenza vaccine viruses. The majority of U.S.-produced influenza vaccines use egg-based manufacturing and viruses adapted for growth in eggs. Amino acid changes in these egg-adapted viruses might contribute to differences in antigenicity from circulating viruses. Although this can occur in all types/subtypes, it was most evident in circulating A(H3N2) viruses, where half showed reduced inhibition by antisera to the egg-adapted vaccine reference virus. Whereas the overall number of circulating B/Victoria viruses was low, a substantial amount of antigenic drift from the vaccine reference virus B/Brisbane/60/2008 was observed.

Interim estimates of the effectiveness of the 2017–18 inactivated influenza vaccines against medically attended respiratory illness published in February 2018 were 36% (95% confidence interval [CI] = 27%–44%) overall, 25% (CI = 13%–36%) against illness caused by influenza A(H3N2) viruses, 67% (CI = 54%–76%) against illness caused by influenza A(H1N1)pdm09, and 42% (CI = 25%–56%) against illness caused by influenza B viruses (*9*). Even during seasons when vaccine effectiveness is reduced, vaccination can offer substantial benefit and reduce the likelihood of severe outcomes, including hospitalization and death. This season’s estimates will be published later this year; however, during the 2016–17 season, vaccination averted an estimated 5.29 million illnesses,^††††^ 2.64 million medical visits, and 84,700 influenza-associated hospitalizations.

The timing of the peaks for certain influenza surveillance indicators this season was unusual. Influenza activity in children typically precedes that in adults, and peak ILI and laboratory positivity percentages precede the peak in hospitalizations, followed by the mortality peak. In this season, influenza-associated hospitalizations and mortality peaked earlier than the percentage of specimens testing positive for influenza in clinical laboratories and the percentage of outpatient visits for ILI. Influenza activity peaked among older adults earlier than among children and young adults; this also occurred, to a lesser extent, during the 2016–17 season (*5*). 

Previous influenza A(H3N2)-predominant seasons have also been associated with increased hospitalizations and deaths; however, the 2017–18 season followed an A(H3N2)-predominant season, and all severity indicators were higher than during the 2016–17 season. The majority of A(H3N2) viruses were genetically characterized as 3C.2a clade, similar, but genetically distinct from the 3C.2a1 subclade that predominated during the 2016–17 season, and from the viruses that circulated during Australia’s 2017 influenza season (*7*,*10*). Outside the United States and Canada, A(H3N2) viruses did not predominate in other Northern hemisphere temperate countries. Further studies are needed to understand the virologic, host, or environmental factors responsible for this high severity season.

The severity of this influenza season highlights the importance of public health measures to control and prevent influenza. Annual influenza vaccination remains the most effective way to prevent influenza illness. Although influenza activity in the United States is typically low during the summer, influenza cases and outbreaks can occur, and clinicians should consider influenza in the differential diagnosis of respiratory illnesses at any time of year. CDC recommends prompt treatment with influenza antiviral medications for persons with confirmed or suspected influenza who are severely ill or at high risk for serious influenza complications. Health care providers should consider novel influenza virus infections in persons with ILI and swine or poultry exposure, or with severe acute respiratory infection after travel to areas where avian influenza viruses have been detected. Providers should alert the local public health department if novel influenza virus infection is suspected. Clinical laboratories using a commercially available influenza diagnostic assay that includes influenza A virus subtype determination should contact their state public health laboratory to facilitate transport and additional testing of any unsubtypeable influenza A–positive specimen. Public health laboratories should immediately send unsubtypeable influenza A viruses to CDC, because early identification and investigation are critical to ensuring timely risk assessment and implementation of appropriate public health measures.

Influenza surveillance reports for the United States are posted online weekly (https://www.cdc.gov/flu/weekly). Additional information regarding influenza viruses, influenza surveillance, influenza vaccine, influenza antiviral medications, and novel influenza A infections in humans is available online (https://www.cdc.gov/flu).

**Summary**

**What is already known about this topic?**

CDC collects, compiles, and analyzes data on influenza activity and viruses in the United States.

**What is added by this report?**

The 2017–18 influenza season was a high severity, A(H3N2)-predominant season. Influenza activity indicators were notable for the volume and intensity of influenza cases that occurred in most of the country at the same time. Record hospitalization rates and high numbers of influenza-associated pediatric deaths also were reported.

**What are the implications for public health practice?**

Receiving a seasonal flu vaccine each year remains the best way to protect against seasonal influenza and its potentially severe consequences. Testing for seasonal influenza viruses and monitoring for novel influenza A virus infections should continue year-round.

## Footnotes

* The CDC influenza surveillance system collects five categories of information from eight data sources: 1) virus surveillance (U.S. World Health Organization collaborating laboratories, the National Respiratory and Enteric Virus Surveillance System (NREVSS), and novel influenza A virus case reporting); 2) outpatient illness surveillance (U.S. Outpatient Influenza-Like Illness Surveillance Network [ILI-Net]); 3) mortality (the National Center for Health Statistics Mortality Surveillance System and influenza-associated pediatric mortality reports); 4) hospitalizations (FluSurv-NET, which includes the Emerging Infections Program and surveillance in three additional states); and 5) summary of the geographic spread of influenza (state and territorial epidemiologist reports). https://www.cdc.gov/flu/weekly/fluactivitysurv.htm.

† Data as of June 1, 2018.

§ The 10 regions include the following jurisdictions: *Region 1*: Connecticut, Maine, Massachusetts, New Hampshire, Rhode Island, and Vermont; *Region 2*: New Jersey, New York, Puerto Rico, and the U.S. Virgin Islands; *Region 3*: Delaware, District of Columbia, Maryland, Pennsylvania, Virginia, and West Virginia; *Region 4*: Alabama, Florida, Georgia, Kentucky, Mississippi, North Carolina, South Carolina, and Tennessee; *Region 5*: Illinois, Indiana, Michigan, Minnesota, Ohio, and Wisconsin; *Region 6*: Arkansas, Louisiana, New Mexico, Oklahoma, and Texas; *Region 7*: Iowa, Kansas, Missouri, and Nebraska; *Region 8*: Colorado, Montana, North Dakota, South Dakota, Utah, and Wyoming; *Region 9*: Arizona, California, Hawaii, Nevada, American Samoa, Commonwealth of the Northern Mariana Islands, Federated States of Micronesia, Guam, Marshall Islands, and Republic of Palau; *Region 10*: Alaska, Idaho, Oregon, and Washington.

¶ Next generation sequencing uses advanced molecular detection to identify gene sequences from each virus in a specimen and thus reveals the genetic variations among many different influenza virus particles in a single specimen; these methods also reveal the entire coding region of the genomes. https://www.cdc.gov/amd/project-summaries/influenza-vaccines.html.

** A virus is considered “reference virus-like” if its hemagglutination inhibition (HI) or neutralization focus reduction assay (FRA) titer is within fourfold of the homologous HI/FRA titer of the reference strain. A virus is considered as low to the reference virus if there is an eightfold or more reduction in the HI or FRA titer when compared with the homologous HI or FRA titer of the reference strain.

†† Defined as a fever (temperature ≥100°F [≥37.8°C], oral or equivalent) and cough or sore throat, without a known cause other than influenza.

§§ The national and regional baselines are the mean percentages of visits for influenza-like illness (ILI) during noninfluenza weeks for the previous 3 seasons plus two standard deviations. Noninfluenza weeks are defined as periods of ≥2 consecutive weeks during which each week accounted for <2% of the season’s total number of specimens that tested positive for influenza. National and regional percentages of patient visits for ILI are weighted based on state population. Use of the national baseline for regional data is not appropriate.

¶¶ Activity levels are based on the percentage of outpatient visits in a jurisdiction attributed to ILI and are compared with the average percentage of ILI visits that occur during weeks with little or no influenza virus circulation. Activity levels range from minimal, corresponding to ILI activity from outpatient clinics at or below the average, to high, corresponding to ILI activity from outpatient clinics much higher than the average. Because the clinical definition of ILI is nonspecific, not all ILI is caused by influenza; however, when combined with laboratory data, the information on ILI activity provides a clearer picture of influenza activity in the United States.

*** For this surveillance component, 54 jurisdictions participate: the 50 states, the District of Columbia, Guam, Puerto Rico, and U.S. Virgin Islands.

††† Levels of activity are 1) no activity; 2) sporadic: isolated laboratory-confirmed influenza cases or a laboratory-confirmed outbreak in one institution, with no increase in activity; 3) local: increased ILI, or two or more institutional outbreaks (ILI or laboratory-confirmed influenza) in one region of the state, with recent laboratory evidence of influenza in that region; virus activity no greater than sporadic in other regions; 4) regional: increased ILI activity or institutional outbreaks (ILI or laboratory-confirmed influenza) in two or more outbreaks, but less than half of the regions in the state with recent laboratory evidence of influenza in those regions; and 5) widespread: increased ILI activity or institutional outbreaks (ILI or laboratory-confirmed influenza) in at least half of the regions in the state, with recent laboratory evidence of influenza in the state.

§§§ FluSurv-NET conducts population-based surveillance for laboratory-confirmed, influenza-associated hospitalizations in children and adolescents aged <18 years (since the 2003–04 influenza season) and adults aged ≥18 years (since the 2005–06 influenza season). FluSurv-NET covers approximately 70 counties in the 10 Emerging Infections Program states (California, Colorado, Connecticut, Georgia, Maryland, Minnesota, New Mexico, New York, Oregon, and Tennessee) and additional Influenza Hospitalization Surveillance Project (IHSP) states. IHSP began during the 2009–10 season to enhance surveillance during the 2009 H1N1 pandemic. IHSP sites included Idaho, Iowa, Michigan, Oklahoma, and South Dakota during the 2009–10 season; Idaho, Michigan, Ohio, Oklahoma, Rhode Island, and Utah during the 2010–11 season; Michigan, Ohio, Rhode Island, and Utah during the 2011–12 season; Iowa, Michigan, Ohio, Rhode Island, and Utah during the 2012–13 season; and Michigan, Ohio, and Utah during the 2013–14, 2014–15, 2015–16, and 2016–17 seasons. Cumulative unadjusted incidence rates are calculated using CDC’s National Center for Health Statistics population estimates for the counties included in the surveillance catchment area. Laboratory confirmation is dependent on clinician-ordered influenza testing, and testing for influenza often is underutilized because of the poor reliability of rapid test results and greater reliance on clinical diagnosis for influenza. Therefore, cases identified as part of influenza hospitalization surveillance likely are an underestimation of the actual number of persons hospitalized with influenza.

¶¶¶ Persons at higher risk include 1) children aged <2 years; 2) adults aged ≥65 years; 3) persons with chronic pulmonary conditions (including asthma), cardiovascular disease (except hypertension alone), renal, hepatic, hematologic (including sickle cell) disease, metabolic disorders (including diabetes mellitus), or neurologic and neurodevelopmental conditions (including disorders of the brain, spinal cord, peripheral nerves, and muscles, such as cerebral palsy, epilepsy [seizure disorders], stroke, intellectual disability [mental retardation], moderate to severe developmental delay, muscular dystrophy, or spinal cord injury); 4) persons with immunosuppression, including that caused by medications or by human immunodeficiency virus infection; 5) women who are pregnant or postpartum (within 2 weeks after delivery); 6) persons aged ≤18 years who are receiving long-term aspirin therapy; 7) American Indians/Alaska Natives; 8) persons with extreme obesity (i.e., body mass index ≥40); and 9) residents of nursing homes and other chronic care facilities.

**** The seasonal baseline proportion of pneumonia and influenza (P&I) deaths is projected using a robust regression procedure, in which a periodic regression model is applied to the observed percentage of deaths from P&I that were reported by the National Center for Health Statistics Mortality Surveillance System during the preceding 5 years. The epidemic threshold is set at 1.645 standard deviations above the seasonal baseline.

††††
https://www.cdc.gov/flu/about/disease/2016-17.htm .

## References

[1] Zhou B, Wentworth DE. Influenza A virus molecular virology techniques. Methods Mol Biol 2012;865:175–92. 10.1007/978-1-61779-621-0_11 22528160

[2] Zhou B, Lin X, Wang W, Universal influenza B virus genomic amplification facilitates sequencing, diagnostics, and reverse genetics. J Clin Microbiol 2014;52:1330–7. 10.1128/JCM.03265-13 24501036 PMC3993638

[3] Davlin SL, Blanton L, Kniss K, Influenza activity—United States, 2015–16 season and composition of the 2016–17 influenza vaccine. MMWR Morb Mortal Wkly Rep 2016;65:567–75. 10.15585/mmwr.mm6522a3 27281364

[4] Appiah GD, Blanton L, D’Mello T, Influenza activity—United States, 2014–15 season and composition of the 2015–16 influenza vaccine. MMWR Morb Mortal Wkly Rep 2015;64:583–90. 26042650 PMC4584770

[5] Blanton L, Alabi N, Mustaquim D, . Update: influenza activity in the United States during the 2016–17 season and composition of the 2017–18 influenza vaccine. MMWR Morb Mortal Wkly Rep 2017;66:668–76. 10.15585/mmwr.mm6625a3 28662019 PMC5687497

[6] Food and Drug Administration Center for Biologics Evaluation and Research. Summary minutes: 151st Vaccines and Related Biological Products Advisory Committee. Silver Spring, MD: US Department of Health and Human Services, Food and Drug Administration; 2018. https://www.fda.gov/downloads/advisorycommittees/committeesmeetingmaterials/bloodvaccinesandotherbiologics/vaccinesandrelatedbiologicalproductsadvisorycommittee/ucm602610.pdf

[7] World Health Organization. Recommended composition of influenza virus vaccines for use in the 2018–2019 northern hemisphere influenza season. Wkly Epidemiol Rec 2018;93:133–41. 29569429

[8] Biggerstaff M, Kniss K, Jernigan DB, Systematic assessment of multiple routine and near real-time indicators to classify the severity of influenza seasons and pandemics in the United States, 2003–2004 through 2015–2016. Am J Epidemiol 2018;187:1040–50. 10.1093/aje/kwx334 29053783 PMC5908755

[9] Flannery B, Chung JR, Belongia EA, Interim estimates of 2017–18 seasonal influenza vaccine effectiveness—United States, February 2018. MMWR Morb Mortal Wkly Rep 2018;67:180–5. 10.15585/mmwr.mm6706a2 29447141 PMC5815489

[10] Sullivan SG, Chilver MB, Carville KS, Low interim influenza vaccine effectiveness, Australia, 1 May to 24 September 2017. Euro Surveill 2017;22. 10.2807/1560-7917.ES.2017.22.43.17-00707 29090681 PMC5718387

